# Adipose Tissues Have Been Overlooked as Players in Prostate Cancer Progression

**DOI:** 10.3390/ijms252212137

**Published:** 2024-11-12

**Authors:** Kia T. Liermann-Wooldrik, Elizabeth A. Kosmacek, Rebecca E. Oberley-Deegan

**Affiliations:** Department of Biochemistry and Molecular Biology, 985870 University of Nebraska Medical Center, Omaha, NE 68198, USA; kliermann@unmc.edu (K.T.L.-W.);

**Keywords:** inflammation, drug therapy, obesity, radiation, oxidative stress, tumor microenvironment, pro-inflammatory

## Abstract

Obesity is a common risk factor in multiple tumor types, including prostate cancer. Obesity has been associated with driving metastasis, therapeutic resistance, and increased mortality. The effect of adipose tissue on the tumor microenvironment is still poorly understood. This review aims to highlight the work conducted in the field of obesity and prostate cancer and bring attention to areas where more research is needed. In this review, we have described key differences between healthy adipose tissues and obese adipose tissues, as they relate to the tumor microenvironment, focusing on mechanisms related to metabolic changes, abnormal adipokine secretion, altered immune cell presence, and heightened oxidative stress as drivers of prostate cancer formation and progression. Interestingly, common treatment options for prostate cancer ignore the adipose tissue located near the site of the tumor. Because of this, we have outlined how excess adipose tissue potentially affects therapeutics’ efficacy, such as androgen deprivation, chemotherapy, and radiation treatment, and identified possible drug targets to increase prostate cancer responsiveness to clinical treatments. Understanding how obesity affects the tumor microenvironment will pave the way for understanding why some prostate cancers become metastatic or treatment-resistant, and why patients experience recurrence.

## 1. Introduction

Prostate cancer is the third most diagnosed cancer in the United States behind only skin cancer and breast cancer [1,2] and is the second most diagnosed cancer in males around the world [3]. A total of 1 in 8 men will be diagnosed with prostate cancer during their lifetime. Of those men, 1 in 41 will die due to complications related to prostate cancer [2,4]. Therapeutic options include surgery, radiation, hormonal-based therapies, and chemotherapy [5]. While the five-year survival rate for prostate cancer is quite high, metastasis, castration resistance, and recurrence result in prostate cancer mortality [2,4]. In a prospective study on men with prostate cancer, adiposity was evaluated and deemed to be associated with characteristics of a higher Gleason Score in patients diagnosed with prostate cancer [6].

Obesity is one of the most common diseases in the world, with 1 in 8 people, or 39.2% of males, living with obesity [7]. Obesity, induced by poor diet, is characterized by large amounts of white adipose tissue (WAT) accumulation [8]. WAT has a large number of lipid droplets, which can aid in the formation of tumors by providing the components needed for metabolism, membrane biogenesis, and signaling molecules. Obesity is also associated with chronic adipose tissue inflammation and oxidative stress that leads to changes in metabolism, immune cell recruitment, and cytokine secretion, which creates a pro-tumorigenic landscape for cancer cells to thrive [9]. Not only is obesity linked to a worse prognosis in prostate cancer, but many other cancers as well, including, endometrial and ovarian cancer, breast cancer, and colon cancer [10,11].

In recent years, there have been a few published papers suggesting that abdominal radiation therapy causes dysregulation of adipokine secretion and changes in the immune system toward favoring tumor growth [12,13,14]. Similarly, some studies have identified that excess adipose tissue in the tumor microenvironment (TME), such as seen in obesity, impacts the efficacy of chemotherapeutic agents and androgen deprivation treatments [15,16]. However, the full impact of aberrant adipose tissue on the TME remains unclear.

This review describes changes in adipose tissue that occur during obesity and aims to provide insight as to how common therapeutic options for prostate cancer are affected by obesity and dysregulated adipocyte signaling, and in turn how the therapeutics affect adipose tissue. Adipocytes affect the TME through cytokines, adipokines, and lipid release. Obesity creates a more oxidatively stressed and pro-inflammatory environment, which promotes tumor initiation and progression. As obesity rates continue to rise, a larger portion of prostate cancer patients will be obese and it is unclear how visceral and subcutaneous adipose tissues and obesity affect standard treatments for prostate cancer such as ADT, radiation, and chemotherapy. The goal of this review is to magnify these areas that need more work carried out to improve prostate cancer patient outcomes.

## 2. Adipose Tissue Composition

In humans, the abdominal regions have two main types of WAT, subcutaneous and visceral fat [17]. Visceral adipose tissue differs from subcutaneous fat in the type of adipocyte, endocrine function, lipolytic activity, and associated inflammatory cells [18].

**Figure 1:**
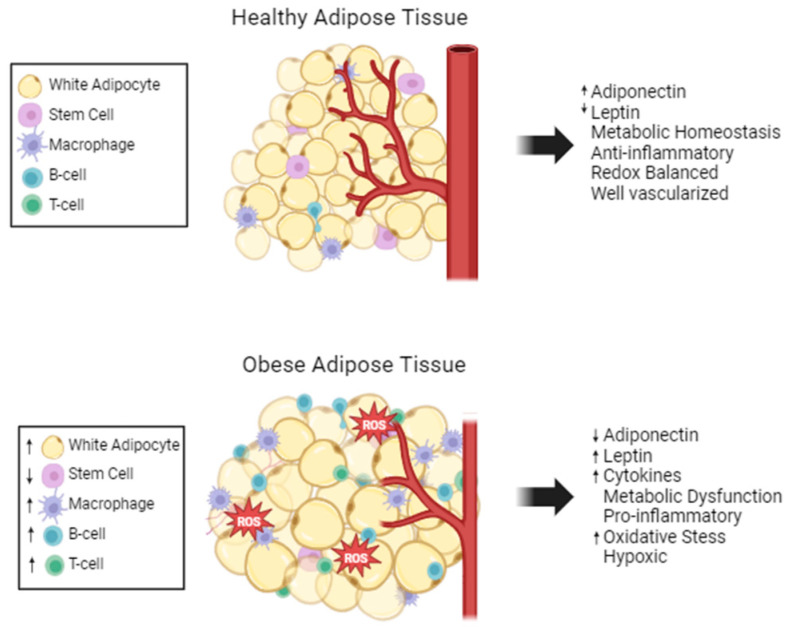
Adipose tissue composition changes with obesity. Healthy adipose tissue is well maintained and regulated. Stem cells and a few anti-inflammatory immune cells are present. Obese adipose tissue has fewer mature adipocytes, but the adipocytes are larger in size. There is excess ROS and limited vascularization in obese adipose tissue. Many pro-inflammatory cells are present, and the metabolism and cytokine secretions are dysregulated, increased (↑) and decreased (↓).

Structurally, WAT is composed of white adipocytes containing one large lipid droplet, which flattens and pushes the nucleus to the edge of the cell, and very few mitochondria. Adipocytes have two main functions, lipid handling and endocrine functions. Besides adipocytes, WAT is comprised of numerous other cell types, such as adipose stem cells (ASCs), immune, stromal, endothelial, and nerve cells; WAT is considered to be well vascularized and innervated (Figure 1) [19]. The complexity of the adipose tissue results in complex systemic signaling through the secretion of adipokines. This complex endocrine signaling allows adipose tissue to maintain energy homeostasis and communicate its nutrient status to the rest of the body.

Within the adipocyte, the large lipid droplet holds fatty acids in the form of triglycerides within the lumen, making the lipid droplet an energy-storing organelle that maintains energy homeostasis. When there is excess energy, fatty acids are converted to acyl-coA, which can then be metabolized into triglycerides and stored within the lipid droplet. Conversely, when energy is needed by cells, fatty acids are mobilized through lipolysis. Lipolysis involves lipase enzymes cleaving triglycerides to free the glycerol head from the three fatty acid tails [20]. Lipolysis is a heavily regulated process [21,22], and once hydrolyzed, free fatty acids are dispersed from the adipocyte to enter the vasculature [23]. Lipogenesis and lipolysis work together to maintain metabolic homeostasis.

As part of the endocrine function of adipose tissue, adipocytes release fatty acid binding protein 4 (FABP4) at the same time lipolysis occurs [24]. FABP4 is a lipid chaperone secreted from adipocytes that regulates glucose homeostasis [25]. Additionally, adipocytes secrete insulin-like growth factor-1 (IGF-1) that stimulates angiogenic growth factors in endothelial cells and ASCs to proliferate and survive [26,27].

The subcutaneous and visceral adipose depots play a crucial role in maintaining energy homeostasis by storing and releasing fatty acids as needed [28]. Within WAT, there is heterogeneity in the capacity to store lipids and the rate of fatty acids release, which has strongly been linked to the location of the adipose tissue [29]. For example, preadipocytes express gene signatures indicative of their depot of origin [30]. Mature adipocytes from the visceral WAT have significant enhancement of pathways related to glucose and lipid metabolism and stress response as compared to abdominal subcutaneous WAT [31]. Functionally, visceral WAT is more metabolically active than subcutaneous WAT [32].

Visceral adipose tissue contains more androgen and adrenergic receptors than subcutaneous fat, which maintains homeostatic lipolysis levels and, in turn, makes visceral adipocytes more metabolically active [33,34]. Additionally, subcutaneous and visceral adipose tissues differ in the synthesis and secretion of adipokines. For example, visceral fat is more heavily associated with pro-inflammatory cytokine secretion.

## 3. Effect of Obesity on Adipose Tissue

Alterations to WAT structure and function are major contributors to many diseases. Adipose tissue changes drastically with obesity. Adipose tissue structure and function are changed by increased adipocyte size and the induction of chronic low-grade inflammation [35]. Hypertrophy of adipocytes is linked to adipose tissue dysfunction and increased risk of metabolic syndromes [36]. In obesity, fat accumulates around organs and in the pelvic region.

The differentiation status of pre-adipocytes found in WAT is determined by metabolic status [37]. In an obese state, visceral WAT has reduced adipogenic capacity and current literature suggests this is due to enhanced inflammation and oxidation, which induces senescence of ASCs [38,39]. An in vitro study by Silva and collaborators noted that obese ASCs secrete more pro-inflammatory cytokines than mature adipocytes or ASCs from lean patients [40]. Additionally, obese ASCs proliferate less and undergo early senescence as compared to non-obese ASCs [41]. Obese adipose tissue is hypoxic due to enlarged adipocytes blocking vascularization. This causes fibroblast activation and excessive accumulation of the extracellular matrix [42]. Activated fibroblasts promote cancer development and progression by secreting factors that induce angiogenesis and immune evasion.

Adipose tissue maintains its immune cell population through the secretion of adipokines. Obesity causes the hypertrophy-induced cell death of adipocytes, which increases monocyte chemoattractant protein 1 (MCP1) expression [43]. Therefore, a hallmark of obesity is dead or dying adipocytes surrounded by macrophages producing crown-like structures [43]. B-cell and T-cell levels are also increased in obese adipose tissue. Another inflammatory marker, C-reactive protein (CRP) is positively correlated with abdominal obesity in a clinical study [44]. CRP modulates the expression of matrix metalloproteinases (MMPs) and MMP inhibitors, which include extracellular matrix remodeling in adipose tissue [45]. Overall, obesity creates a vicious cycle where adipose tissue structure is altered leading to increased inflammation and oxidative stress, and the inflammation and oxidative stress causes further dysregulation of adipocyte structure and function (Figure 1).

Oxidative stress is another distinct feature of obesity [46]. Reactive oxygen species (ROS) are highly reactive molecules that when elevated damage proteins, lipids, and DNA. High levels of ROS are linked to dysfunctional cellular homeostasis and tissue damage [47,48]. Calcium exchange, dysregulated by obesity-associated hyperlipidemia, leads to mitochondrial Ca^2+^ overload and subsequently increases mitochondrial ROS production [49]. Additionally, obese patients have less glutathione peroxidase 3 (GPx3), a hydrogen peroxide scavenger, in their adipose tissues [50]. GPx3 expression is attenuated by high levels of tumor necrosis factor-α (TNFα) and hypoxia and depressed levels lead to a rise in systemic oxidative stress and increased occurrence of metabolic complications [50]. An activated immune system further feeds the obesity–oxidative stress cycle, creating increased ROS production while dampening antioxidant capabilities.

Even in a normal, healthy state, adipocytes are vulnerable to oxidative stress. Due to the abundance of lipids found in the membrane and lipid droplets of adipocytes, adipose tissue is highly susceptible to lipid peroxidation [51]. Lipid peroxidation is generally described as the process in which oxidants attack lipids and convert them into peroxide and hydroperoxide derivatives [52]. These lipid peroxides can undergo further reactions to modify DNA and proteins and induce more oxidative damage. Carbon–carbon double bond lipids, such as polyunsaturated fatty acids (PUFAs), are prone to lipid peroxidation [51]. Mature adipocytes have reduced DNA repair mechanisms making these cells more vulnerable to DNA oxidation, as well [53].

## 4. Obesity and Prostate Cancer

A characteristic of cancer progression is the epithelial-to-mesenchymal transition (EMT), which enables cancer cells to leave the primary TME. During EMT, E-cadherin is downregulated, and N-cadherin and vimentin are upregulated loosening cell–cell contacts and resulting in a migratory phenotype [54]. Obesity enhances EMT in cancer cells. Specifically, prostate cancer cells cultured in serum from obese and normal-weight mice showed that obese serum induced EMT [55], supporting the idea that obesity enhances cancer cell invasiveness. A possible mechanism is that adipose tissue releases the chemokine (C-X-C motif) ligand 12, CXCL12, otherwise known as stromal cell-derived factor 12. CXCL12 enhances migration through the activation of STAT3, NF-κB, and MAPK signaling pathways [56,57]. CXCL12 and its receptor, chemokine (C-X-C motif) receptor 4 (CXCR4), are found in high concentrations in the stromal compartment of prostate cancer tumors [58,59]. The use of a CXCR4 antagonistic blocker, AMD3100, or CRISPR-Cas9 knockout of CXCR4 decreased the metastatic potential of prostate cancer cells [59]. In addition, the chemokine (C-C motif) ligand 7 (CCL7) is another factor that directs the migration of prostate cancer cells harboring its respective receptor, C-C chemokine receptor type 3 (CCR3), which is linked to poor prognosis [60]. Obese adipocytes release more CCL7 increasing this migratory signaling pathway when compared to normal adipocytes [60].

Along with CXCL12/CXCR4 and CCL7/CCR3 signaling, FABPs and peroxisome proliferator-activated receptors (PPARs) are established biomarkers for malignant prostate cancers; elevated expression levels of both FABPs and PPARs are strongly correlated with higher Gleason scores [61,62]. FABP4^+^ prostate cancer cells were significantly more invasive as compared to cells treated with a FABP inhibitor, and treatment with a fatty acid, oleic acid, was able to further enhance the invasiveness of FABP4^+^ prostate cancer cells [63]. FABP and fatty acids secreted from adipocytes in the TME aid in the ability of cancer cells to invade tissue by providing energy to cancer cells. PPAR-γ is highly expressed in adipose tissue where it plays a role in differentiating preadipocytes into mature adipocytes [64]. PPAR-γ mRNA and protein expression are elevated in prostate cancer biopsies [65]. PUFAs activate PPAR-γ, which in turn upregulates vascular endothelial growth factor (VEGF), aiding in the creation of a blood supply, rich with nutrients, within the TME [65]. VEGF also acts upon the Akt3 serine/threonine kinase to retain the PPAR-γ coactivator 1a (PGC1a) in the nucleus, leading to mitochondrial biogenesis and switching to a primarily oxidative form of cellular metabolism [66]. Increasing electron transport chain activity provides cancer cells with more energy to proliferate and migrate to other areas of the body [66]. Obesity leads to the increased presence of free fatty acids, chemokines, and cytokines that can activate cellular pathways, such as PPAR-γ, which participate in high-energy processes allowing for cancer cells to thrive.

## 5. Adipose Tissue Effects on Cancer

### 5.1. Adipose-Regulated Prostate Cancer Metabolism

#### 5.1.1. Adipose as an Energy Source for Cancer Cells

In patients with prostate cancer, those with increased peri-prostatic fat are more likely to experience relapsed cancer [67]. The metabolic flux of prostate cancer is dynamic and provides the framework for migration and proliferation and is affected by lipids in the TME. In the early stages, prostate cancer cells rely on glycolysis, but when cells become castration-resistant, they rely more on oxidative phosphorylation. As the cells metastasize, they utilize oxidative phosphorylation, glycolysis, and β-oxidation, increasing the reliance on free fatty acids [68,69]. Also, prostate cancer cells dysregulate their lipid metabolism to increase the production of proteins needed for de novo lipogenesis [70,71,72]. Apart from the de novo synthesis, excess adipose tissue supplies TME of breast cancer and leukemia cells with free fatty acids, adipokines, and immune cells to stimulate the cancer cells to proliferate and metastasize to distant organs and aid in the signaling process of many biological pathways, such as anti-apoptosis [73,74]. The vascularization of WAT keeps a rich supply of nutrients entering the adipose tissue, which can be secreted to cancer cells to help drive proliferation and metastasis.

The TME and oxygen levels have known effects on prostate cancer metabolism [75]. Prostate cancer’s hypoxic environment promotes lipid accumulation [76]. A portion of the TME consists of adipocytes [77,78]. Prostate cancer cells overexpress fatty acid synthase protein (FASN) and monoacylglycerol lipase (MAGL), both metabolic enzymes that promote the formation and breakdown of lipids, which are now thought to be metabolic oncogenes [79,80,81]. Both in vitro and in vivo models confirmed that FASN and MAGL, in the presence of FABP5, enhance the metastatic potential of prostate cancer [82,83]. Specifically, FABP5 interacts with lipases to control the efflux of fatty acids from adipocytes by driving lipolysis [84], and the increased availability of fatty acids provides cancer cells with the energy needed to sustain continuous movement through the cell cycle.

Since adipocytes are a major contributor to the TME, there is an assumed large number of fatty acids available for energy consumption in prostate cancer cells. Fatty acids are broken down by the β-oxidation metabolic pathway, which connects to other pathways synthesizing metabolic products such as ATP, acetyl-coA, and steroid hormones. Gene analysis of prostate cancer cells revealed increased expression of branched-chain fatty acid β-oxidation enzymes that break down long-chain fatty acids in the peroxisome [85].

Another regulator of prostate cancer metabolism is the mTOR pathway [86,87], which serves as a hub for the integration of metabolism, cell growth, and disease [88,89]. Activation of mTOR mediates the activity of sterol regulatory element-binding protein (SREBP1), a master regulator of lipid metabolism [90]. SREBP1 binds transcriptional start sites of de novo fatty acid synthesis genes and recruits transcriptional machinery to enhance expression [91]. Along with elevated expression of FASN, stearoyl-CoA dehydrogenase (SCD) also exhibited increased expression when SREBP1 was overexpressed in prostate cancer [92,93]. Many cancer cells overexpress SCD to avoid lipotoxicity by maintaining homeostasis between lipid synthesis and lipid oxidation [94,95]. Silencing SCD would disrupt the homeostatic nature of the TME making it a potential therapeutic target [93,96].

#### 5.1.2. Adipose as a Source of Building Blocks for Proteins in Cancer Cells

Many cancer cells utilize amino acids, nucleic acids, and lipids from their environment to maintain the necessary molecules required for proliferation [97,98,99]. To metabolize these molecules into the oxidized biomass precursors, nicotinamide adenine dinucleotide (NAD+) is needed to maintain metabolic pathways such as glycolysis, TCA cycle, and oxidative phosphorylation [100]. Limited availability of NAD+ decreases the accessibility of biomass substrates and cellular proliferation both in vitro and in vivo [101,102,103]. Alternatively, the synthesis of highly unsaturated fatty acids (HUFA) from PUFA aids in the regeneration of NAD+; thus, helping the cell to maintain a proliferative state [104]. During hypoxia, the de novo synthesis of fatty acids is repressed while the exogenous uptake of lipids is increased [105]. The increased availability of lipids leads to the activation of fatty acid β-oxidation pathways that help to maintain the NAD+/NADH pool needed for the synthesis of biomolecules, such as proteins.

Several groups have identified that the lipid-rich environment surrounding the tumor results in the upregulation of fatty acid β-oxidation in prostate cancer cells [106,107,108]. This metabolic phenotype of prostate cancer makes the inhibition of fatty acid β-oxidation an interesting target for limiting tumor growth and sensitizing cells to hormone-based therapies such as enzalutamide [108,109]. Nasser and co-workers identified 2,4 dienoyl-CoA reductase (DECR1), a rate-limiting enzyme in the β-oxidation of PUFAs, as being upregulated in patient prostate cancer samples and correlated with shorter relapse-free survival rates [110]. Knockdown of DECR1 in androgen receptor sensitive and resistant cell lines resulted in decreased cell viability, colony formation, invasion, and migration [110], suggesting a plausible therapeutic target of β-oxidation. The presence of lipids in TME provides the tools for cancer cells to maintain energy and biomolecule levels needed for rapid proliferation.

#### 5.1.3. Adipose as a Source for Maintaining Membranes in Cancer Cells

Metabolically, lipids are needed for more than just energy; they are needed to help maintain cellular composition. There are three main lipid classes found in the plasma membrane: glycerophospholipids, sphingolipids, and sterols [111,112]. In mammalian cells, cholesterol is the main plasma membrane sterol and is synthesized using the mevalonate pathway of isoprenoid metabolism [113]. Lipids are first converted to acetyl-CoA by β-oxidation. In the mevalonate pathway, the addition of two acetyl-CoAs leads to the formation of 3-hydroxy-3-methylglutaryl-CoA (HMG-CoA), which is formed into sterols including cholesterol to be used to form new membranes. Two mevalonate pathway enzymes, HMG-CoA synthase 1 and HMG-CoA reductase, are overexpressed in prostate cancer, especially at the early stage [114]. Studies have shown that inhibition of the mevalonate pathway, using statin drugs, enhances tumor suppression [115,116,117]. In prostate cancer, blocking the formation of cholesterol with statins induces apoptosis [114,118].

In cancer, membrane lipids are often abnormal in that they contain a lower amount of oxidizable fatty acids, increasing resistance to oxidative damage and chemotherapy treatments [112]. Cellular membranes are comprised of phospholipids, which are synthesized in the endoplasmic reticulum through the process of fusing glycerol-3-phosphate and fatty acyl-CoA [119]. Hobby et al. discovered that exogenous fatty acids can supply the fatty acyl chains needed for membrane biosynthesis [120], establishing the idea that adipocytes secrete fatty acids for cancer cells to take up and utilize for membrane biosynthesis.

#### 5.1.4. Obesity Effects on Metabolism, Building Blocks, and Membranes

Obesity is a risk factor for many metabolic diseases due to the development of insulin resistance. Elevated free fatty acid levels are the primary cause of insulin resistance, as are dysregulated adipokines and oxidative stress [121]. In the late 1990s, fatty acids were found to inhibit the action of insulin by dampening the signaling propagation from insulin receptors [122,123]. Decreased insulin signaling in adipose tissue results in increased lipolysis and elevated free fatty acid release [124]. The mitochondria of adipocytes are dysregulated in an obese state, and genes involved in fatty acid synthesis, uptake, and β-oxidation are upregulated [125,126]. Augmentation of mitochondrial energy homeostasis, through decreased fatty acid oxidation, leads to cellular dysfunction and lipotoxicity by synthesizing excessive levels of fatty acids [127,128].

In obesity, the accumulation of ROS byproducts leads to impaired translation and an enhanced ribosomal stress response [129]. Impairment of NAD+ synthesis affects the homeostatic nature of protein, carbohydrate, and lipid metabolism contributing to both the development of obesity and the resulting complications [130]. In adipose tissue where the availability of lipids is high, oxidative conditions lead to the production of reactive aldehydes such as trans-4-hydroxy-2-nonenal (4-HNE) through stress-induced lipid peroxidation [53]. 4-HNE in adipose tissue increases protein carbonylation, which is the ROS-related oxidation of protein sidechains [131]. The same study identified adipose-specific FABP as being carbonylated, which results in reduced activity. Thus, increased protein carbonylation is correlated with increased occurrence of metabolic disorders during obesity [132].

While the availability of lipids is needed for the biosynthesis of cellular membranes, the accumulation of lipids, like in obesity, affects this biosynthesis. Deficiency in enzymes related to phospholipid synthesis protects mice from diet-induced obesity [133], and abnormally high or low concentrations of phospholipids alter glucose and lipid metabolism [134]. Increased exogenous saturated fatty acid uptake decreases membrane fluidity [135]. Decreased fluidity is caused by ROS linked to metabolic dysfunction in obesity [136]. Therefore, in obesity, the excess accumulation of lipids allows for membrane biosynthesis to continue and new adipocytes to be synthesized.

Obese adipose tissue is highly vascularized. The vascularization of WAT keeps a rich supply of nutrients entering the adipose tissue, which can be secreted to cancer cells to help drive proliferation and metastasis. The notable innervation of WAT provides a pathway for cancer cells to enter the adipose tissue through a process called perineural invasion (PNI) [137]. This is another way in which cancer cells are able to utilize adipose tissue.

### 5.2. Adipokine Signaling and Cancer Progression

Obesity dysregulates adipocyte metabolism systemically leading to the aberrant release of adipokines. Adipokines released from adipose tissue, such as leptin and adiponectin, play an active role in the proliferation of prostate cancer cells. Since a large population of the world is considered obese, there has been a vast amount of research conducted on the effect of obesity on adipokine secretion, specifically leptin, adiponectin, visfatin, apelin, and resistin. Leptin is an adipocyte-specific hormone that when released systemically, activates the hypothalamus, the brain region responsible for satiety, to control appetite and energy expenditure [138,139]. Adiponectin, also secreted from adipocytes, has been established to regulate energy homeostasis through lipid and carbohydrate metabolism and exert anti-inflammatory effects [140]. Leptin is greatly upregulated, and adiponectin is downregulated during obesity [141]. Not only is leptin upregulated in patients with obesity, but high leptin expression is also correlated to poor prognosis in several cancers, including thyroid, ovarian, prostate, and breast [141,142,143,144]. One study elucidated the antagonistic effects of adiponectin and leptin on carcinogenesis and discovered adiponectin was able to inhibit cell migration, invasion, and proliferation, while increased leptin levels induced protumorigenic properties, such as increased proliferation and migration [141]. Overall, increased amounts of leptin have been attributed to tumorigenic characteristics while decreased amounts of adiponectin are tied to a lack of activated anti-tumor pathways playing an influential role in the crosstalk between dysregulated adipose tissue and cancer.

#### 5.2.1. Adipokine Secretion Is Associated with Cancer Cell Progression

Leptin is an upstream activator of JNK and causes proliferation in an androgen-independent prostate cancer cell [145]. During obesity, leptin expression is increased, which shortens the G1 phase by promoting the transcription of cyclin D1, the regulator of the transition from the G1 to S phase [146]. The expression of cyclin D1 activates cyclin-dependent kinase 4/6 (CDK4/6), which results in quick entry to the S phase, resulting in faster DNA replication [147]. Leptin and its receptor, Ob-R, are expressed in the hypothalamus, human vasculature, and endothelial cells [138,148]. In vivo, corneal vascularization was observed in WT but not in Ob-R deficient rats [148], substantiating the notion that an increased leptin expression could enable cancer cells to grow blood vessels. In gastric and ovarian cancers, leptin can induce migration and decrease cell-to-cell adhesion by activating the JAK-STAT and MEK pathways [149,150]. In vitro studies using intestinal epithelial cells identified leptin as a contributing factor in lipid droplet formation through the mTOR pathway, a prominent cellular growth pathway [151]. In many cancers, the mTOR pathway is upregulated, possibly due to the dysregulated presence of leptin [152]. The observed results of leptin have not been characterized in prostate cancer; however, given the high correlation between obesity and prostate cancer, it is assumed that leptin will have similar effects on prostate TME.

Visfatin, also known as nicotinamide phosphoribosyl transferase (NAMPT), is an adipokine secreted from visceral adipose tissue. Visfatin is the rate-limiting enzyme in the biosynthesis of NAD, which plays a role in cellular homeostasis and maintaining cell viability [153]. Visfatin is upregulated in the serum of patients with different types of cancer including prostate, endometrial, and liver [154,155,156,157]. In the context of cancer, visfatin has been found to promote malignancy through increasing angiogenesis via VEGF [155], mediating chemotherapy resistance, and inducing cellular proliferation.

Other adipokines that have been shown to play a role in prostate cancer progression include apelin and resistin. Apelin is a ligand to the apelin receptor (APJ) and is expressed by both the prostate and adipose tissue [158]. Through the APJ axis, apelin downregulates tissue inhibitors of matrix metalloproteinases (TIMP2) expression to increase prostate cancer progression and metastasis [159,160]. In support of this study, elevated apelin expression in the serum of cancer patients has been reported [158]. Additionally, the adipokine resistin induces prostate cancer migration, invasion, and proliferation [161,162].

#### 5.2.2. Adiponectin Is Associated with Anti-Tumor Properties

Adiponectin inhibits cell migration, invasion, and proliferation and has many anti-inflammatory effects. Mice with diet-induced obesity were given exogenous adiponectin leading to decreases in colon cancer lesion formation as compared to mice with obesity that were not given adiponectin [163]. This same study determined that adiponectin inhibits mTOR through adenosine monophosphate-dependent kinase (AMPK) activation, both of which control cell growth and metabolism [163]. Disulfide bond A oxidoreductase-like protein (DsbA-L) regulates adiponectin multimerization in 3T3 cells and, thus, adiponectin activity [164,165]. High molecular weight adiponectin is the most biologically active form, mediating insulin sensitivity and glucose homeostasis [166]. Deficiency of DsbA-L not only leads to the misfolding and subsequent removal of adiponectin through the ER-associated degradation (ERAD) pathway [164], but it also suppresses the production of interferon-γ, a cytokine with important anti-tumor effects [167].

AMPK is a known repressor of mTOR [168,169]. Reduced levels of adiponectin result in unchecked mTOR regulation and enhanced lipid metabolism, which together promote cancer proliferation and progression. Thus, the lack of adiponectin caused by obesity plays an active role in cancer. This effect is potentially working with insulin resistance and subsequent hyperinsulinemia often found in obese patients. Insulin is a signaling hormone in the body that is released from pancreatic beta cells when blood glucose levels are high triggering the activation of enzymes associated with glycolysis and glycogenesis and blocking fatty acid oxidation [170]. Insulin resistance, therefore, promotes the metabolism of fatty acids providing cancer cells with the building blocks needed for proliferation. Additionally, adiponectin is a positive regulator of apoptosis [171,172]. Thus, decreased adiponectin levels promote the proliferation of cancer cells by limiting apoptotic cell death.

### 5.3. Adipose Tissue Inflammation and Cancer Progression

**Figure 2:**
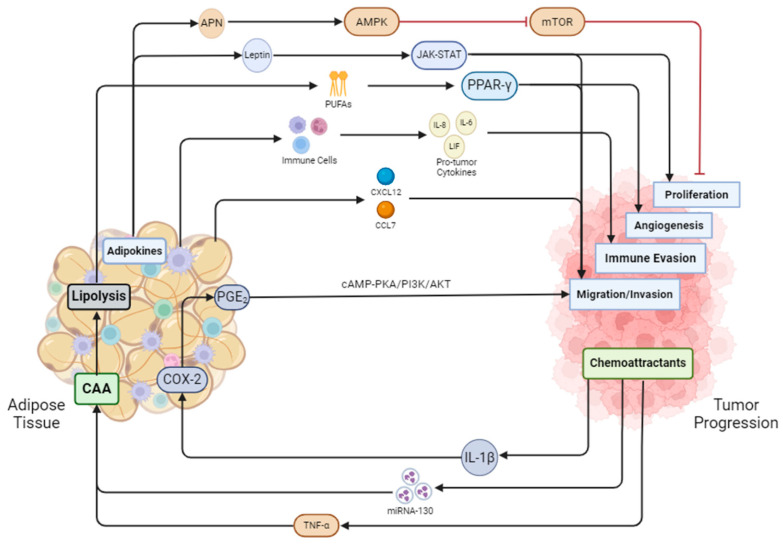
Adipose tissue and cancer are in constant crosstalk with one another. Adiponectin secreted by adipose tissue inhibits mTOR and in turn blocks proliferation, while leptin activates the JAK-STAT pathway to promote proliferation. Adipose tissue undergoes lipolysis secreting fatty acids that activate PPAR-γ, initiating angiogenesis in tumor cells. Immune cells from adipose tissue secrete pro-tumor factors aiding the tumor in evading the immune system. Adipose tissue also secretes cytokines such as CXCL12 and CCL7 to induce prostate cancer cell migration. Prostate cancer also releases cytokines such as IL-1B, which activates COX-2 and PGE2. PGE2 then cycles back to the tumor to promote migration and invasion through the cAMP-PKA/PI3K/AKT pathway. MiRNA-130 and TNF-α from cancer cells promote the dedifferentiation of adipocytes into cancer-associated adipocytes (CAAs). CAAs undergo enhanced lipolysis, feeding the cancer cells fatty acids.

Over the past decade, many reviews have suggested that adipocytes are a major regulator of the immune system. However, very few studies have linked the complexity of the adipose tissue environment to the TME, specifically in terms of the immune cell composition and how it regulates the tumor’s activity. Co-culturing of adipocytes and cancer cells leads to the upregulation of many pro-inflammatory cytokines such as family members of interleukin-6 (IL-6), a multifunctional cytokine in host defense, and CXCL1, which is a chemoattractant for many immune cells, including neutrophils [173,174]. These same cytokines also polarize macrophages toward an anti-inflammatory, M2 phenotype, which is responsible for tissue healing, or, in the context of cancer, the creation of a favorable TME [175]. With obesity-related inflamed adipose tissue, other aberrantly regulated adipokines aid in the recruitment of immune cells and the survival of cancer cells by activating proliferation and cellular growth pathways [173,174,176]. Most studies investigating the link between adipose tissue and cancer have been conducted in the context of breast cancer. However, since obesity is one of the leading risk factors for prostate cancer, chronic inflammation in the adipose tissue is possibly one factor aiding in the creation of prostate TME [6,9]. Local inflammation is a shared quality in both obese and cancer patients. This crosstalk between the tumor and the adipose tissue and vice versa provides many avenues to explore how secreted proteins found within, surrounding, and distant from the primary tumor affect progression and metastasis (Figure 2).

#### 5.3.1. Adipocyte Secreted Cytokines and Tumor Progression

Adipose tissue surrounding the tumor and surrounding the organs that contain tumors play a unique role in tumor formation and progression. Obese adipose tissue is rich in T cells, being the second most numerous leukocyte population in adipose tissue behind macrophages [177]. T cells in adipose tissue release leukemia inhibitory factor (LIF), a cytokine from the IL-6 family, which controls stem cell self-renewal, inflammatory response, and cancer progression [178,179]. LIF induces breast cancer migration through the activation of the ERK1/STAT pathway [173]. CXCL1, CXCL2, and CXCL3 are chemo-attractants released by tumor cells that stimulate white adipose tissue to release adipose stromal cells, as well as activate the ERK/STAT/LIF pathway [173,174]. Inhibition of these pathways leads to reduced metastatic and angiogenic properties of cancer cells [180]. In obese prostate cancer patients, not only is there an abundance of WAT, but CXCL1 is upregulated in epithelial cells giving more opportunity for the movement of adipocytes to the TME [174]. This suggests that blocking the CXCL family could potentially reduce the ability of prostate cancer to metastasize.

Another chemoattractant that plays a significant role in tumor progression is interleukin-8 (IL-8) secretion. Lipid droplet accumulation in adipocytes leads to the release of IL-8, which consequently is significantly upregulated in obesity [181]. In metastatic breast cancer cells, IL-8 is upregulated compared to the primary breast cancer cells [182], causing pro-angiogenic effects [183]. In another study, in vitro and in vivo models confirmed that IL-6 is overexpressed in adipose tissue that surrounds breast cancer tumors. Additionally, higher adipocyte IL-6 expression correlates with worse prognosis [184], and increased total body adipose tissue corresponds to increased levels of serum IL-6 [185,186]. In patients, elevated levels of IL-6 are more likely to be produced from omental adipose tissue as compared to subcutaneous tissues, where most fat deposits reside in obesity [186]. IL-6 signaling also plays a role in the polarization of macrophages and the upstream activation of interleukin-4, which is responsible for adipose tissue macrophage proliferation [187], as well as regulating metabolic homeostasis [186]. Thus, adipocyte-derived IL-6 has a multifaceted role in the TME.

#### 5.3.2. Macrophages

Macrophages are a group of diverse myeloid cells that are a part of the innate immune response and clear dead cells and pathogens [188]. In normal tissue remodeling, macrophages can recognize the presence of phosphatidylserine on the extracellular side of the plasma membrane, which is a signal for the removal of apoptotic cells through phagocytosis [189,190]. However, macrophages are extremely plastic cells and can change phenotypically when exposed to certain stimuli [191,192]. There are two well-established classifications for macrophages: classically activated (M1) and alternatively activated [188,193,194]. M1 macrophages are characterized as being pro-inflammatory and M2 macrophages are considered anti-inflammatory [192,195]. However, this is an oversimplification and there are other classifications of macrophages beyond M1 and M2.

In obesity, the number of macrophages in the adipose tissue is often very high causing chronic, local inflammation [196]. Obesity causes decreased adiponectin levels leading to adipocyte death, which stimulates the macrophages in the adipose tissue to polarize toward a pro-inflammatory phenotype [195] to enhance the influx of bone marrow-derived monocyte progenitor cells differentiating into macrophages [197]. In creating an inflammatory environment, macrophages secrete cytokines, such as TNFα, IL-6, and IL-8, which spread throughout the body creating chronic, systemic inflammation [197]. This is how the chronic inflammation of obese adipose tissue is established and sustained.

Tumor-associated macrophages (TAMs) found within the TME have a phenotype that is not M1 or M2 but rather an ability to transition between the two [176]. TAMs are speculated to aid in tumor progression by using the M1 phenotype to infiltrate the TME [198]. Once the macrophages are in the TME, colony-stimulating factor (CSF-1) is released from the tumor cells to trigger the phenotypic change of the M1 macrophages to M2 [199,200]. The M2 phenotype is special in its ability to promote tumor cell proliferation and invasion by secreting factors, such as epithelial growth factor and TGF-β, as well as increasing the activation of pathways such as MAPK [193,200,201]. In an in vivo model using diphtheria toxin to deplete macrophages, it was established that macrophages are needed for angiogenesis to occur, further solidifying the need for macrophages in the creation and maintenance of the TME [176]. In patients with ovarian cancer, the presence of M2-like macrophages was strongly correlated with a worse prognosis [202], solidifying the idea that an aim for cancer therapies could be to block the phenotypic switching through CSF-1 inhibitors.

Macrophages isolated from adipose tissue contain excessive amounts of lipid droplets driving inflammation and altering the metabolism of the neighboring cells [203]. TAMs from breast cancer tumors contain increased expression of FABPs [204]. Macrophages collected from a mouse lung metastasis contained an abundance of lipid droplets [193]. Likewise, macrophages co-cultured with tumor cells had a higher accumulation of lipids than macrophages cultured with normal cells. Interestingly, macrophages co-cultured with tumors had similar levels of lipids observed in adipose tissue-associated macrophages [191,203]. In an analysis of primary and metastatic samples taken from patients with prostate cancer, a cluster of FABP genes was significantly upregulated in the metastatic samples [205], supporting the idea that fatty acid utilization is not only needed at the primary tumor but also during metastasis. Such high expression levels of lipids in the TAMs suggest that obesity could accelerate the formation of tumors by recruiting macrophages containing energy-filled lipid droplets to the TME. In conclusion, macrophages from obese adipose tissue drive tumor formation and cancer progression.

### 5.4. Oxidative Stress from Obesity Drives Cancer Progression

Another hallmark of obesity is increased oxidative stress. The overabundant supply of energy substrates in obesity is thought to drive mitochondrial dysfunction and ROS signaling [206]. While ROS normally function as signaling molecules, if uncontrolled, they can damage DNA, lipids, and proteins. Since adipocytes are full of triglyceride lipids, adipose tissue is especially vulnerable to the effects of oxidative stress, which results in adipose tissue remodeling, aberrant signal transduction, and heightened inflammation [207].

Prostate cancer exposure to an adipocyte-rich environment, in vitro and in vivo, induced the upregulation of heme-oxygenase (HO-1), an oxidative stress enzyme [208]. Increased HO-1 levels can increase the metastatic potential, as well as promote growth and invasiveness in prostate cancer cells [208,209]. Paracrine signaling by periprostatic adipose tissue (PPAT) is upregulated in obesity. Laurent et al. elucidated that free fatty acids released by the PPAT stimulate the expression of NADPH oxidase 5 (NOX5) in prostate cancer cells residing nearest PPAT [210]. NOX5 expression plays a role in inducing proliferation and survival in prostate cancer [211]. Additionally, NOX5 is implicated in activating HIF-1α in normoxic conditions [212], and obesity induces a signaling cascade in adipocytes that leads to the activation of HIF-1α [213]. In prostate cancer, HIF-1α functions to drive angiogenesis, cell proliferation, and metastasis [214].

Decades ago, prostate cancer tumorigenesis was linked to increased levels of ROS and an impaired ability to maintain redox homeostasis. Abundant ROS induces spontaneous mutagenesis by damaging cellular DNA [215,216,217], suggesting a possible mechanism for ROS-mediated tumorigenesis. In addition, the presence of lipid peroxidation products such as malondialdehyde and acrolein in plasma and tumor tissues can form DNA adducts, which have been positively correlated with prostate cancer progression [218]. Obesity accompanied by oxidative stress drives DNA mutation and dysregulated signaling pathways, supporting cancer progression.

## 6. Cancer Effects on Adipose Tissue

Both tumor cells and adipocytes secrete proteins and small molecules that function as signaling molecules. Tumor cells can secrete cytokines that recruit adipose stromal cells to the microenvironment, and on the other hand, adipocytes can secrete adipokines that aid in the development of the TME [174,182]. Tumor cells can attract nearby adipocytes by releasing TNF-α and exosomes containing miRNA-130, both of which inhibit the transcription of PPAR-γ, leading to the dedifferentiation of mature adipocytes into cancer-associated adipocytes (CAAs) [208,219]. CAAs provide the tumor with fatty acids for energy requirements, lipids for membrane biosynthesis, and protect the growing tumor from immune infiltration. Cancer cells and adipocytes are in constant crosstalk with one another to modulate the expression of proteins, cytokines, and other cells within the TME [220] (Figure 2).

Prostate cancer is known to metastasize to regions rich in adipocytes, such as bone marrow. Bone marrow adipocyte numbers increase with age and obesity, both of which are risk factors for metastatic cancer [221]. As previously stated in this review, lipids derived from adipose tissue can help supply cancer cells with the energy needed to maintain proliferation. Alternatively, cancer cells can trigger the activation of lipolysis pathways to increase the release of lipids from adipocytes [222].

Herron et al. determined that IL-1β secreted from prostate cancer cells interacts with bone marrow adipocytes to increase the expression of cyclooxygenase-2 (COX-2), an enzyme needed to form prostaglandin E_2_ (PGE_2_), promoting both survival and proliferation of metastatic cells [223,224]. In prostate cancer, PGE_2_ phosphorylates STAT3, which induces a mesenchymal phenotype [225] and promotes invasion via the cAMP-PKA/PI3K-Akt signaling pathway [226]. At the molecular level, few studies have elucidated the effects that cancer formation and progression have on surrounding adipose tissue and adipocytes.

From a clinical standpoint, the indirect impact of cancer on adipose tissue is well documented and described. The treatment of prostate cancer can require the use of chemotherapeutics. Such treatments are known to affect adipose tissue composition and distribution within the human body [227]. Another study found that the peri-prostatic adipose tissue area is increased in patients who develop castration-resistant prostate cancer (CRPC) [228], suggesting that the development of CRPC alters the adipose distribution in the abdominal cavity. Vertulli et al. found a strong correlation between recurrence after radical prostatectomy and levels of abdominal adipose tissue [229]. Overall, these findings suggest that incomplete removal of prostate tumors in a patient with heightened adiposity can increase the risk of recurrence in the same manner that adipose tissue can induce primary tumor formation.

## 7. Cancer Treatments Cause Changes in Adipocytes

### 7.1. Hormone-Based Therapy: ADT

Many prostate cancer tumors express androgen receptors (ARs) and prostate-specific androgens. Hormonal-based therapies, such as androgen deprivation therapies (ADT), are frequently used in prostate cancer to reduce the number of androgens circulating throughout the prostate gland to reduce prostate cancer growth [230,231]. Although most prostate cancers initially respond to ADT, long-term treatment results in a cell population that can survive in low levels of androgen [232]. More and more studies demonstrate that lipids play a role in castration resistance.

Clinical studies show that the use of ADT is associated with changes to adipose tissue composition, specifically increased fat mass [233,234]. Such an increase often leads to a second co-morbidity, obesity, which as shown in this review and others, increases the risk of prostate cancer progression. While the connection between prostate cancer, ADT, and obesity has been well established in the clinical setting, less can be said about the impact of the three at the molecular level. For example, ADT increases body fat mass, the risk of insulin resistance, and serum adiponectin levels [235,236]. In one study, periprostatic fat collected from patients who received six months of ADT treatments showed a significant trend of pro-inflammatory, obesity-like adipose tissue [236]. Differential tissue composition and gene-enrichment analysis pointed toward the infiltration of immune cells in the adipose tissue following ADT treatment [237]. Taken with the notion that obesity is a risk factor for prostate cancer, ADT could potentially be causing adverse effects on the adipose tissue linking it to tumor progression after treatment.

Testosterone is the main androgen that is repressed in prostate cancer patients who receive ADT. Testosterone is a type of steroid hormone that regulates male and female sexual characteristics, maintains the strength of bones and muscles, and stimulates erythropoiesis [238]. The synthesis of testosterone starts with the oxidative cleavage of lipid cholesterol and ends with the androstenedione being reduced by 17β-hydroxysteroid dehydrogenase to yield testosterone [239]. Decreased testosterone is associated with an increase in total cholesterol levels and low-density lipoprotein (LDL) [240], both are positively correlated to an increased risk for incidence and progression of cancer [241,242,243]. Elevated cholesterol is important for protein transport and for the synthesis of new membranes [244,245], and elevated LDL levels can cause an increase in sensitivity to oxidative stress [246].

**Figure 3:**
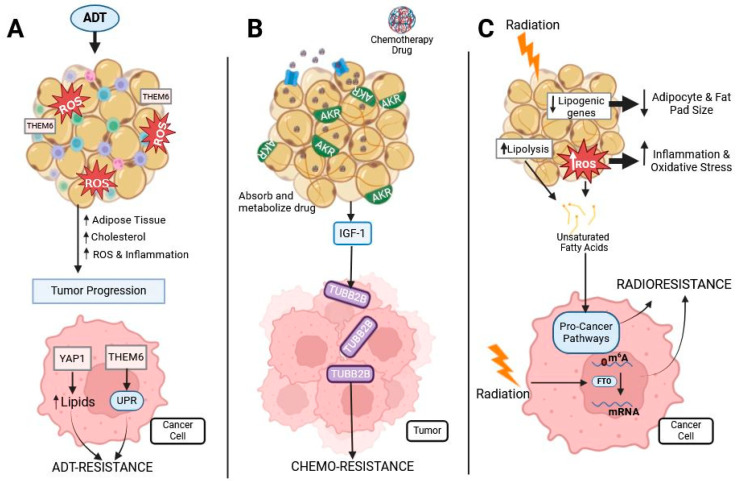
Prostate cancer treatments’ effect on adipose and cancer. Prostate cancer is commonly treated with ADT, chemotherapies, and radiotherapy, all of which interact with adipose tissue: (**A**) ADT treatment induces oxidative stress and inflammation in adipose tissue. This causes an increase in adipose tissue, cholesterol, oxidative stress, and inflammation, leading to tumor progression. Obesity-related proteins THEM6 and YAP1 are associated with ADT resistance in prostate cancer by increasing cellular lipid content and activating the UPR. (**B**) Chemotherapy is often lipophilic and, therefore, easily sequestered and metabolized by adipocytes containing the AKR protein, reducing the availability of the drug for prostate tumors. Chemotherapy induces dysregulated cytokine secretion from adipose tissue, such as an increased release of IGF-1, causing the upregulation of TUBB2B, which is associated with chemo-resistance. (**C**) Radiation to adipose tissue decreases lipogenic gene expression and increases ROS and lipolysis. Both ROS and lipolysis lead to the release of unsaturated fatty acids from the adipocytes, which cancer cells use to activate pro-cancer pathways. Additionally, radiation activates FTO, which demethylates mRNA to promote radioresistance.

Thioesterase superfamily member 6 (THEM6), which is highly expressed in adipose tissue where it regulates intracellular fatty acid homeostasis, is upregulated in ADT-resistant prostate cancers, and activates the unfolded protein response (UPR) [247]. Blomme et al. found that depletion of THEM6 leads to lipid remodeling in cancer cells, particularly the loss of ether triglycerides, and re-sensitizes prostate cancer cells to ADT [247]. Additionally, yes-associated protein 1 (YAP1) is found to be overexpressed in enzalutamide-, an ADT drug, resistant prostate cancers [248,249]. YAP1 maintains ADT resistance by contributing to cancer stemness and lipid metabolism, in which YAP1^+/+^, ADT-resistant cells had high levels of free fatty acids and increased expression of genes related to lipid metabolism compared to YAP1^−/−^, ADT-resistant cells [250]. Both THEM6 and YAP1 are proteins that regulate lipids and are intertwined with ADT resistance at a molecular level (Figure 3A).

### 7.2. Chemotherapy

As stated above, obesity has been linked to poor outcomes in patients with multiple different cancers [6,10,11], but the exact mechanism is unknown. Clinically, increased adiposity is positively associated with worse survival in men undergoing ADT and docetaxel treatment [251,252]. Adipocytes are known to sequester lipophilic molecules [253]. Since most chemotherapeutic agents are hydrophobic, they are readily taken up by adipocytes, which contributes to the development of chemoresistance (Figure 3B). Additionally, in vitro and in vivo studies demonstrate that adipocytes secrete factors that induce chemo-resistance in prostate cancer [254,255].

Some of the mechanisms through which adipocytes are believed to induce chemotherapeutic resistance are by metabolizing drugs and altering the redox status of the cancer cells. Cancer cells that express high levels of CD36, a fatty acid transporter, have a more drug-resistant profile than cancer cells that do not express CD36 [256], suggesting a potential interplay between fatty acid metabolism and drug resistance. A pivotal study from 2017, provides strong evidence that adipocytes can absorb and metabolize daunorubicin, a chemotherapeutic drug commonly used to treat leukemias, altering its pharmacokinetics [257]. Adipocytes express an abundant amount of aldo-keto reductase (AKR) enzymes [257,258]. Additionally, AKR is expressed at higher levels in obese individuals and is thought to play a role in obesity-associated chemoresistance [257]. AKRs metabolize many chemotherapeutics including anthracyclines, mitomycin, cisplatins, antitubulin agents, vinca alkaloids, and cyclophosphamides [259]. From this study, we can infer that obese subjects will have less chemotherapeutic efficacy, which is one possible mechanism for obesity-related chemotherapeutic resistance. Abundant adipose tissue and lipids not only promote pro-tumor properties but also disrupt the treatment of cancers leading to chemoresistance and increased likelihood of recurrence. Thus, there is a need to better understand the role that adipocytes have on the TME (Figure 3B).

In prostate cancer, docetaxel is the first line of defense for patients with CRPC [260]. Insulin-like growth factor-1 (IGF-1) secreted from peri-prostatic fat significantly increases prostate cancer cell expression of TUBB2B, β-tubulin isoform 2B [261]. TUBB2B is known to be upregulated in highly metastatic prostate cancer and promotes docetaxel resistance by preventing drug-induced microtubule stabilization [262]. Cabazitaxel is a chemotherapeutic commonly prescribed to metastatic CRPC patients. Like docetaxel, cabazitaxel binds to tubulins impairing microtubule formation, blocking mitosis, and initiating apoptosis [263]. In the presence of ASCs, prostate cancer cells were chemo-resistant to docetaxel and cabazitaxel [255]. Mechanistically, ASCs decrease ROS levels found within the TME, reducing the effect of chemotherapeutic drugs [255]. Therefore, depletion of ASCs from tumors may re-sensitize cancer cells to chemotherapy (Figure 3B).

### 7.3. Radiotherapy

Another common treatment option for those with prostate cancer is external beam radiation therapy (EBRT). EBRT uses high-energy X-rays or electron beams to kill cancer cells by damaging DNA and hindering the ability to replicate [264]. At the cellular level, radiation plays both a direct and an indirect role in subduing DNA replication processes. Radiation can directly cause DNA damage through single- and double-stranded breaks and with the formation of adducts to certain bases [265,266]. Indirectly, the release of radiation energy generates free radicals and ROS through water ionization hours to days after exposure. Increased ROS increases the susceptibility of DNA, protein, and lipid damage [266,267,268]. It has been documented that obese men with prostate cancer are more likely to experience treatment failure following radiotherapy. However, the effects of radiation on adipose tissue are not well understood and have not been extensively described.

When mice receive pelvic radiation, their fat pads atrophy, which correlates to an increase in fibrosis, and skin damage [269,270]. Poglio et al. are one of very few papers outlining the effect of radiation on adipose tissue [271]. Using both sublethal and lethal doses of radiation, they observed pronounced morphological changes to subcutaneous adipose tissue. Within 7 days after radiation exposure, adipocytes begin apoptosis, and a decrease in the mean size of adipocytes is observed. Not only were the mature adipocytes affected by the radiation, but the stromal vascular fraction (SVF) containing adipocyte precursors was damaged [271], and the stroma of adipose tissue underwent rapid extracellular matrix remodeling [272]. Following radiation treatment, the SVF exhibited decreased proliferative capacity and inhibited differentiation potential. Oxidative stress in adipocytes was observed in a dose-dependent manner, through the increase in NOX expression and decrease in manganese superoxide dismutase activity [271], suggesting a link between oxidative stress and adipocyte cell death.

Metabolically, radiation disrupts the usual metabolic profile of cancer cells. In 2011, Jo et al. aimed to elucidate the effects of whole-body radiation on lipogenic-related gene expression in gonadal white adipose tissue in mice [13]. The expression levels of lipogenic genes such as FASN, acetyl-CoA carboxylase, and glucose-6-pyruvate dehydrogenase decreased following radiation suggesting a possible reason for a decreased fat pad size following radiation [13]. After radiation, cancer cells can be found to have increased lipogenic substrates and intracellular unsaturated fatty acids [273,274]. Unsaturated fatty acids are used within the cells to maintain membrane fluidity, decrease the secretion of pro-inflammatory cytokines, improve the function of vascular endothelial cells, and inhibit pro-inflammatory processes [275]. In patient samples, lipogenic pathways have been found to be enriched in those with glioblastoma recurrence following radiation treatment [273]. The increased biosynthesis of free fatty acids protects cells against ER stress and subsequent apoptosis [273], protecting the cells from the damage inflicted by radiation therapy. These metabolic alterations driven by lipids are one way cancer cells can evade radiotherapy leading to higher recurrence rates.

On the protein level, ROS levels can determine protein activation and inhibition through post-translational adducts. Therefore, radiation-induced ROS levels can have a dominant role in cellular signaling. Fat mass and obesity-associated (FTO) protein, whose expression is elevated in obese subjects, is known to play a prominent role in human obesity, energy homeostasis, and pre-adipogenic regulation [276,277], as well as being upregulated in certain cancers [278,279,280]. FTO is an RNA N6-methyladenosine (m^6^A) demethylase that regulates the amount of m^6^A RNA present in nuclear DNA [276,281]. In the presence of radiation, FTO removes m^6^A RNA methylation leading to the promotion of radiotherapy-resistant cancers by blocking ferroptosis potentially through the demethylation of ubiquitin thioesterase, suggesting a possible synergistic mechanism for radiation resistance in obese patients [279,282,283,284] (Figure 3C).

The demonstration of the effects of radiation on the TME has implicated radiation as a culprit in the recurrence of several cancers including gliomas, breast, and thyroid cancers. Radiation damage to adipose tissue also persists years after the initial radiation treatment as documented in a study that followed adult childhood cancer survivors [14]. The same study was able to establish striking similarities in adipose tissue dysfunction between irradiated and obese adipose tissues including activation of inflammatory pathways and altered adipokines [14]. Irradiating the brain, which contains virtually no triglycerides but vast amounts of lipid-derived cholesterol, with 20 Gy of radiation prior to implantation with human xenograft glioblastomas, significantly increases tumor burden and leads to faster mortality due to alteration in metabolic profiles that favored the proliferation of cancer cells such as rises in ATP/GTP and a decrease in antioxidants [274], suggesting that oxidative stress induced through radiation alters lipid-rich microenvironments to promote tumor progression. Another explanation for radiation-induced cancer recurrence is through the enhancement of cancer stem cells following sublethal doses of radiation and subsequent re-initiation of tumors [285,286,287]. Furthermore, persistent remodeling of the extracellular matrix following radiation of adipose tissue could enable the creation of a pro-tumorigenic microenvironment [272]. However, this is an understudied area, and more work needs to be performed to determine the effect radiation has on adipocytes and how this contributes to the TME.

Adipose tissue and lipids play a multifaceted role in promoting tumor progression. After ADT and radiation, adipose tissue becomes inflamed and oxidatively stressed, like obese adipose tissue. Thus, some therapies may induce a pro-tumor microenvironment. Additionally, adipose tissue sequesters and metabolizes lipophilic chemotherapies, reducing efficacy in tumors with an adipose-rich landscape. On the other hand, obese adipose tissue signals to tumor cells to increase proteins and other factors to protect against ADT, chemotherapy, and radiation (Figure 3). Overall, more research into the effect of therapeutics on adipose tissue is needed to fully elucidate the effect that dysregulated adipose tissue can have on the TME and tumor progression.

### 7.4. Strategies to Target Adipose Tissue

Obesity drives prostate cancer progression through the presence of chronic inflammation, aberrant adipokine section, increased ROS, and elevated whole-body fatty acid release. The presence of excess adipose tissue also alters the common treatment options for prostate cancer. To combat the progression of cancer, these pathways related to obesity and adipocytes need to be targeted.

Disrupting lipid metabolism within the TME decreases the availability of free fatty acids that the tumor uses to maintain energy, create membranes, and as signaling molecules. SCD converts saturated fatty acids into monosaturated fatty acids and promotes cancer cell proliferation, migration, and metastasis [94,95]. The SCD inhibitor, CAY10566, has been proven to be effective at reducing oleate levels and blocking the growth of tumors [288]. Blocking the circulation of cholesterol using statins, cholesterol-lowering drugs, may help decrease the formation of new membranes, and thus, reduce the ability of cancer cells to proliferate [114,118]. To decrease the uptake of fatty acids by the cancer cells, CD36 could be targeted and blocked. Sulfo-N-hydroxysuccinimidyl (NHS) ester of oleate (SSO) irreversibly binds CD36 and may work as a drug treatment for cancers [289]. To reduce the rate of β-oxidation, targeting DECR1 has been shown to decrease cell viability, migration, and colony formation [110]. Overall, inhibiting any aspect of lipid metabolism may work to reduce the progression of prostate cancer.

Other potential targets for reducing adipose-related cancer progression include altering adipokine secretions. Obese adipose tissue secretes increased leptin and decreased adiponectin. The pro-tumor qualities induced by leptin would be minimized by inhibiting leptin secretion. In breast cancer, LCF1, a leptin activity inhibitor, decreases cancer cell growth and motility [290]. On the other hand, increasing adiponectin, using metformin, enhances anti-tumorigenic properties [291,292]. Therefore, using LCF1 or metformin in combination with other chemotherapeutic agents may be beneficial in lowering overall tumor burden.

Obesity reduces the efficiency of common therapeutic practices to work on prostate cancer. Both ADT and radiation treatments increase the oxidative stress within adipocytes. Adipocytes need to maintain low levels of oxidative stress to function properly. Therefore, the reduction in ROS within adipose tissue specifically would minimize changes in metabolism, inflammation, and cytokine secretion [9]. Vitamin E scavenges lipid peroxyl radicals, so targeting vitamin E directly to the adipose tissue may help reduce damages associated with excess ROS. Additionally, MnTE-2-PyP (T2E), a manganese porphyrin mimetic, protects fat from radiation damage by scavenging ROS [293]. Within the tumor, excess lipid peroxides induce ferroptosis; however, in the presence of radiation, FTO blocks ferroptosis, promoting radiation resistance [279]. Currently, Bisantrene (CS1) and Brequinar (CS2) are two small-molecule FTO inhibitors shown to reduce RNA methyltransferase activity in acute myeloid leukemia patients but have not been tested in prostate cancer [281].

In prostate cancer, THEM6 and YAP1 play integral roles in contributing to ADT resistance. Depleting THEM6 activity re-sensitizes prostate cancer cells to ADT and induces lipid remodeling [247]. YAP1 inhibition in tumors reduces cancer stemness and minimizes lipid metabolism. Blocking both YAP1 and THEM6 proteins re-sensitizes prostate cancer cells to ADT treatment, and therefore, may be a viable option for CRPCs. Docetaxel is the most prescribed chemotherapy for patients with CRPC [260]. TUBB2B prevents drug-induced microtubule stabilization [262]. Thus, depletion or inhibition of TUBB2B from cancer cells, potentially by minimizing IGF-1 secretion, reduces docetaxel resistance.

Finally, since obesity is strongly correlated with an increased risk of developing cancer, weight loss is thought to be a powerful tool for minimizing the chances of developing cancer. Glucagon-like peptide 1 (GLP-1) receptor agonists, like Ozempic, are prescription medications for patients with type 2 diabetes, heart disease, and more recently, obesity. GLP-1 receptor agonists work by stimulating insulin production and reducing blood sugar levels, promoting weight loss. The current literature is divided on how GLP-1 receptor agonist usage is associated with the risk of cancer development. Some studies have noted that GLP-1 agonist usage increased the risk of thyroid cancers [294,295], while others have demonstrated a decreased risk of prostate cancer progression [296,297]. This is an area in which more research is needed.

Table 1 systematically lays out targets to decrease prostate cancer progression, the potential ways to target those biomolecules, and the impact they have on cancer cells (Table 1).

## 8. Conclusions and Future Perspectives

It is well documented that obese individuals are at a greater risk of developing cancers, including prostate cancer. Current research has shown that adipose tissue and adipocytes play an intimate role in the progression of prostate cancer. In obese adipose tissue, numerous pro-inflammatory cytokines, as well as abundant amounts of ROS are present and secreted into the microenvironment nearby, altering the function of the adipose tissue [44]. Through many reported mechanisms, obese adipocytes induce the progression of prostate cancer by supplying the cancer cells with building blocks necessary for proliferation, migration, and overall survival [183]. Adipocytes secrete adipokines such as leptin, adiponectin, and CCL2 that recruit pro-inflammatory cells to the microenvironment, helping cancer cells evade the immune system [141]. Conversely, the formation of cancer can also drive inflammation in adipose tissue triggering cancer progression [222].

Obese patients do not respond to prostate cancer therapy very well and have higher mortality rates as compared to non-obese prostate cancer patients. Following radiotherapy, obese cancer patients more frequently report urinary and erectile dysfunction compared to their non-obese counterparts [298]. Additionally, the use of combination treatments was found to exacerbate poor quality of life symptoms [299]. It is currently not known why this occurs. In this review, we have demonstrated that adipose tissue can contribute to resistance to standard treatments used in prostate cancer, such as ADT, chemotherapy, and radiation. Elucidating the cellular and molecular mechanisms of adipose-related ADT- and chemo-resistance, and the effect of radiation on large depots of adipocytes, could improve the standard treatment options for those with prostate cancer.

## Figures and Tables

**Table 1:** Potential ways to target adipose-driven pathways in prostate cancer.

Target	Potential Ways to Target	Impact on Cancer Cells
Lipid Release	Stearoyl-CoA dehydrogenase (SCD) inhibitor (CAY10566)	Disrupts lipid homeostasis in tumor microenvironment
Cholesterol	Statin treatment	Blocks formation of cholesterol-inducing apoptosis and inhibits membrane formation
Lipid Uptake	CD36 Inhibitor (SSO)	Blocks the uptake of fatty acids by the cancer cells
Fatty Acid β-oxidation	2,4 Dienoyl-CoA reductase (DECR1) inhibitor	Blocks oxidation of PUFAs inducing cancer cell death and decreasing colony formation, invasion, and migration
Leptin Secretion	Inhibit Leptin secretion	Block pro-tumorigenic properties induced by leptin
Adiponectin Secretion	Increase Adiponectin signaling (metformin)	Induce anti-tumor qualities
Adipose Oxidative Stress	Decrease ROS levels (Vitamin E, T2E)	Reduce signaling cascades induced by oxidative stress and suppress the chances of ROS-derived mutations
Blocking Ferroptosis	FTO inhibitor (Bisantrene, Brequinar)	Maintain m^6^A RNA methylation status
Intracellular Lipid Trafficking	THEM6 inhibitor	Induces lipid remodeling and re-sensitizes prostate cancer cells to ADT
Lipid Metabolism	YAP inhibitor	Reduce cancer stemness and enzalutamide resistance
Microtubule Stabilization	TUBB2B inhibitor	Block docetaxel-resistance
Obesity	GLP-1 Receptor Agonists (Ozempic)	Decrease proliferation
